# Accuracy of Digital and Conventional Full-Arch Impressions in Patients: An Update

**DOI:** 10.3390/jcm9030688

**Published:** 2020-03-04

**Authors:** Alexander Schmidt, Leona Klussmann, Bernd Wöstmann, Maximiliane Amelie Schlenz

**Affiliations:** Dental Clinic-Department of Prosthodontics, Justus Liebig University, Schlangenzahl 14, 35392 Giessen, Germany; leona.klussmann@dentist.med.uni-giessen.de (L.K.); bernd.woestmann@dentist.med.uni-giessen.de (B.W.); maximiliane.a.schlenz@dentist.med.uni-giessen.de (M.A.S.)

**Keywords:** clinical study, digital dentistry, dental impression technique, full arch impression, dimensional measurement accuracy, intraoral scanner

## Abstract

The aim of this clinical study was to update the available data in the literature regarding the transfer accuracy (trueness/precision) of four current intraoral scanners (IOS) equipped with the latest software versions and to compare these data with conventional impressions (CVI). A metallic reference aid served as a reference dataset. Four digital impressions (Trios3Cart, Trios3Pod, Trios4Pod, and Primescan) and one CVI were investigated in five patients. Scan data were analyzed using three-dimensional analysis software and conventional models using a coordinate measurement machine. The transfer accuracy between the reference aid and the impression methods were compared. Differences with *p* < 0.05 were considered to be statistically significant. Overall, mean ± standard deviation (SD) transfer accuracy ranged from 24.6 ± 17.7 µm (CVI) to 204.5 ± 182.1 µm (Trios3Pod). The Primescan yielded the lowest deviation for digital impressions (33.8 ± 31.5 µm), followed by Trios4Pod (65.2 ± 52.9 µm), Trios3Cart (84.7 ± 120.3 µm), and Trios3Pod. Within the limitations of this study, current IOS equipped with the latest software versions demonstrated less deviation for short-span distances compared with the conventional impression technique. However, for long-span distances, the conventional impression technique provided the lowest deviation. Overall, currently available IOS systems demonstrated improvement regarding transfer accuracy of full-arch scans in patients.

## 1. Introduction

In recent years, digitalization has gained increasing importance in daily dental practice [1]. In particular, the workflow for fixed dental prostheses (FDPs) (i.e., digital impression taking and computer-aided design/computer-aided manufacturing) has been further developed [2,3]. Several studies have reported comparable or even higher accuracy for intraoral scans (IOS) compared with conventional impressions for short-span FDPs up to a quadrant [4,5,6,7,8]. For full-arch scans, still a higher transfer accuracy has been described for conventional impression (CVI) techniques [9,10,11]. Several clinical studies investigated the accuracy of full-arch scans for different IOS systems using a conventional impression or a plaster model as reference [12,13,14,15,16]. However, for a precise evaluation of the transfer accuracy, an external reference structure is indispensable [17]. Even anterior structures have already been recorded using an extraoral scanner [18]; the entire jaw of a real patient cannot be scanned using a high precision laboratory scanner to obtain a reference dataset. To overcome these limitations, two independent clinical studies developed a reference aid for the investigation of the transfer accuracy of full-arch scans under clinical conditions [10,17]. They reported, for the first time, real deviations in digital and CVI techniques considering patient movement, saliva, and lack of space [10,17].

Since our last study in 2016 [10], in which we investigated the transfer accuracy of different IOS systems, hardware and software versions have undergone continuous improvement [19]. Thus, actual digital devices presumably further minimize transfer errors between the intraoral environment and the model cast even more as some authors have indicated an improvement of the matching-algorithms [20]. Typical sources of error have been described for IOS systems that may influence scanning accuracy: aside from hardware and software components of the IOS system itself [21,22], scanning path [23,24,25], lack of calibration [26], and user(s) [27] are also potential sources of influence, which is particularly relevant for digital full-arch impressions.

Therefore, the aim of this clinical study was to update the available data in the literature regarding the transfer accuracy of four current IOS systems equipped with the latest software versions and compare these data with conventional impressions. The null hypothesis was that there is no statistical difference in transfer accuracy (trueness and precision, ISO 5725 [28]) between the investigated actual digital systems and a conventional full-arch impression.

## 2. Materials and Methods

Five patients with a complete lower dental arch were included in the present clinical study. The investigation was conducted in full accordance with ethical principles, including the World Medical Association Declaration of Helsinki. This study was approved by the local Ethics Committee of the Justus Liebig University (Giessen, Germany; Ref. no. 163/15, 03.09.2015). To ensure comparable test results, all experiments were performed by a single operator (L.K.) with experience in all impression techniques.

**Figure 1:**
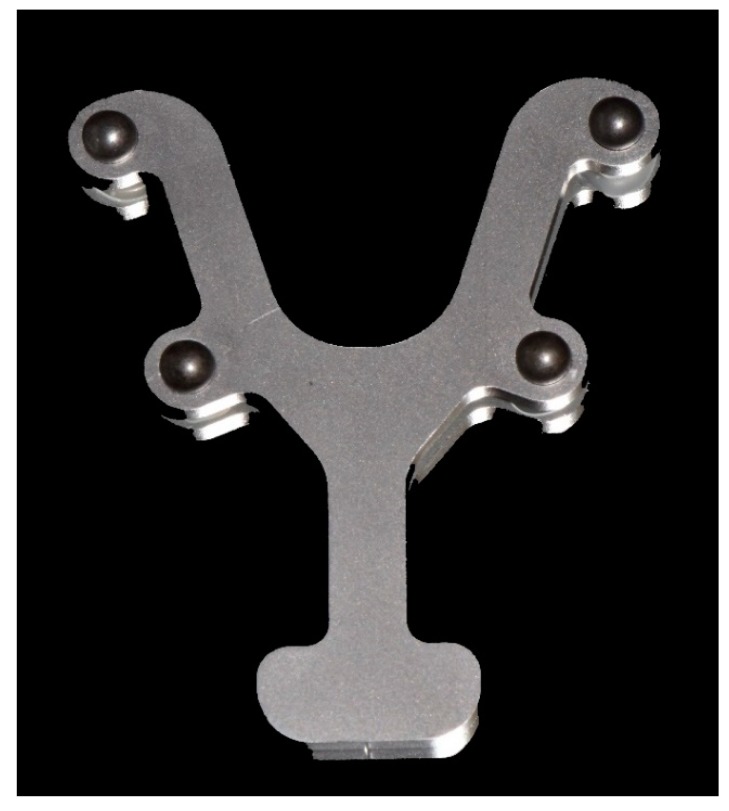
Metallic reference aid with four steel spheres.

According to a method previously described by Kuhr et al. [10], four bearing steel spheres (1.3505 100Cr6 DIN5401; TIS GmbH, Gauting, Germany) with a diameter of 5 mm were reversibly luted on the teeth of the lower jaw using a flowable composite (Grandio Flow, Voco, Cuxhaven, Germany). For exact positioning of the spheres, a metallic reference aid (Bretthauer GmbH, Dillenburg, Germany; Figure 1) [10] was used and the lips and cheeks were retracted using a cheek retractor (Optragate, Ivoclar Vivadent, Schaan, Lichtenstein). 

Subsequently, in every patient four digital full-arch impressions were taken using the Trios 3 Cart wired (“T3CARw”’, version 19.2.4, normal scan speed mode, manufactured 2016-03, 3Shape, Copenhagen, Denmark), the Trios 3 Pod wired (“T3PODw”, version 19.2.4, normal scan speed mode, manufactured 2017-12, 3Shape, Copenhagen, Denmark), the Trios 4 Pod wireless (“T4PODwl”, version 19.2.4, normal scan speed mode, manufactured 2019-08, 3Shape, Copenhagen, Denmark), and the Primescan (“PRI”, version 5.0.1, manufactured 2019-04, Dentsply Sirona, Bensheim, Germany) beginning with the occlusal surface, followed by the oral surface, and returning to the buccal surface [23]. To obtain the best possible scan result under standardized conditions, the IOS systems were calibrated using the respective calibration device according to manufacturer’s instructions. The scan data were directly exported in a standard tessellation language (STL) dataset. After removing the cheek retractor, a CVI was taken using a medium body polyether impression material (Impregum Penta Soft Quick, batch no. 4811262, 3M Espe, Minneapolis, MN, USA) and a standard metal tray (Ehricke stainless steel, Orbis Dental, Münster, Germany). Before casting with type IV dental stone (Fujirock EP, batch no. 1810031, GC Corporation, Tokyo, Japan), the polyether impression was stored for at least 2 h to ensure elastic recovery. The plaster models were stored under laboratory conditions (temperature, 23°C ± 1°C and humidity 50 ± 10%) for a minimum of 5 days. 

**Figure 2:**
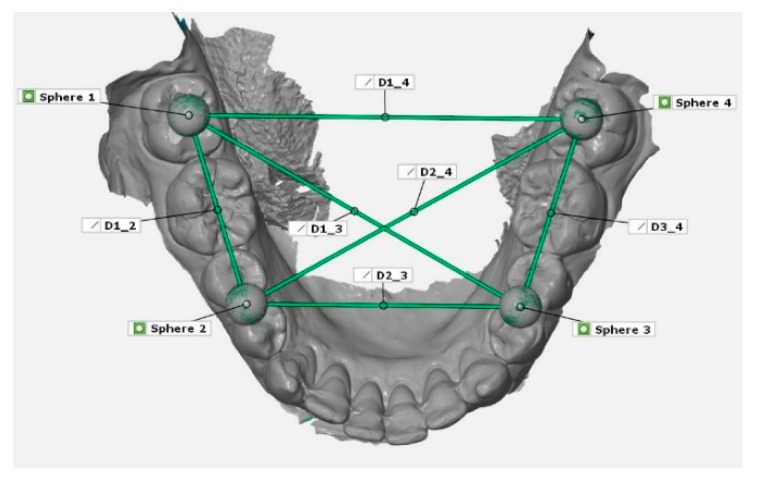
Example of the measurement of linear distances (D1_2, D1_3, D1_4, D2_3, D2_4, D3_4) between centers of the four spheres (1–4).

For measurements of the reference aid and the plaster models, a coordinate measurement machine (CMM; Thome Präzision GmbH, Messel, Germany) with corresponding software (X4 V10 GA x64, Metrologic Group, Meylan, France) was used. To receive a reference dataset, the reference aid with the inserted spheres were measured ten times and mean value for each sphere position were calculated. This digital reference model was stored as a dataset in Initial Graphics Exchange Specification (IGES) format. Thereafter, the spheres of the plaster models of the conventional impressions were also measured with CMM and saved as digital datasets. The STL datasets of the digital impressions were imported into a three-dimensional analysis program (GOM Inspect 2019, Gom GmbH, Braunschweig, Germany) for linear measurement in between the centers of the spheres (1–4; Figure 2). Therefore, the reference dataset of the reference aid was imported and saved as CAD-data in the analysis program. The imported STL dataset was saved as actual data. Within the scan dataset, four spheres were constructed using fitting elements (Gauss best fit, 3 Sigma) according to the scanned spheres. Subsequently, the deviations between the measured distances in the scan datasets and the reference aid were calculated. For surface superimpositions, the spheres of the actual data were assigned to the according CAD-spheres. Thereafter, a prealignment via three-point alignment was applied. For final alignment, a best fit algorithm for superimposition were used. Therefore, it was possible to visualize three-dimensional deviations via surface comparison on CAD data and to examine the deviations of the spheres in y-, x-, and z-directions.

Statistical analysis was performed using SPSS version 25 (IBM Corporation, Armonk, NY, USA). The data were tested for normal distribution (Kolmogorov–Smirnov Test) and variance homogeneity (Levene Test). As variance heterogeneity was present in some of the distances, for robust statistics, the data was corrected according to Games Howell. This procedure is based on Bland and Altman, who recommends parametric calculation in this specific case [29]. According to ISO 5725-1, mean values for the deviations between the IOS results and the reference aid describing trueness, standard deviation describing precision for the different impression techniques were shown [28]. Therefore, Welch-ANOVAS were calculated for each distance (Trueness). Furthermore, the standard deviations were compared with each other via Levene Test (Precision). Differences with *p* < 0.05 were considered to be statistically significant. 

## 3. Results

**Figure 3:**
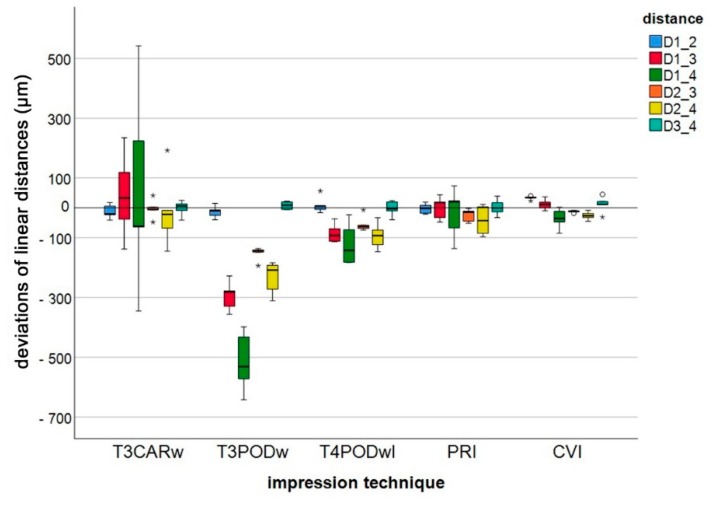
Boxplot diagram of the deviations of linear distances (D1_2, D1_3, D1_4, D2_3, D2_4, D3_4) measured between the centers of the four spheres (1–4) for the different impression techniques (Trios 3 Cart wired (T3CARw), Trios 3 Pod wired (T3PODw), Trios 4 Pod wireless (T4PODwl), Primescan (PRI), conventional impression (CVI); outliers (○), extreme values (*)).

**Figure 4:**
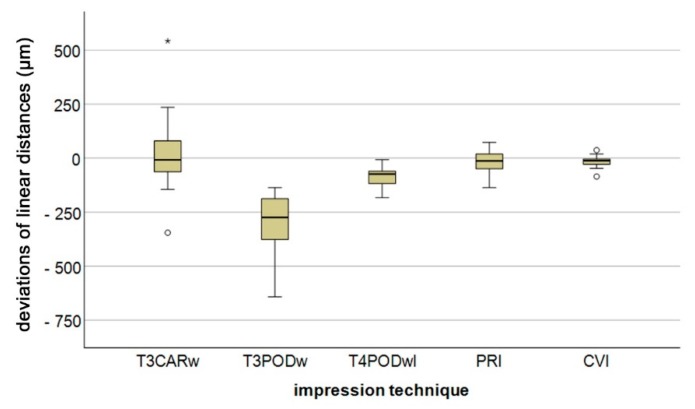
Boxplot diagram of long distance pooled data (except linear distance D1_2 and D3_4) of the deviations of linear distances for the different impression techniques (Trios 3 Cart wired (T3CARw), Trios 3 Pod wired (T3PODw), Trios 4 Pod wireless (T4PODwl), Primescan (PRI), conventional impression (CVI); outliers (○), extreme values (*)).

Calculated differences between the measurement parameters of the five different impression techniques, along with reference values, are reported in Figure 3. For a better overview, pooled data for long distances (except D1_2 and D3_4) is depicted in Figure 4. Regardless of the impression technique, the largest deviation in linear differences was observed for D1_4 (intermolar distance). Except for D1_2 and D3_4, CVI demonstrated the lowest deviation of all impression techniques, with a minimum deviation of 12.3 ± 3.8 µm (D2_3) and a maximum deviation of 36.3 ± 32.6 µm (D1_4). Especially with regard to the short linear distances (D1_2 and D3_4), all IOS systems demonstrated lower deviation compared with CVI, whereas for long distances, CVI still showed the smallest deviations. The greatest deviation was observed for T3PODw, with a minimum deviation of 12.4 ± 8.0 µm (D3_4) and a maximum deviation of 515.0 ± 100.3 µm (D1_4).

Concerning trueness, for the short linear distance (D1_2), only PRI showed significantly better results than CVI. Whereas for all other differences, the investigated digital impression methods were not significantly better than the CVI (*p* < 0.05). For the precision, no significant difference between the five impression techniques was observed for the short linear distances (D1_2 and D3_4). For the long distances, significant differences were found. For a better overview, amount data for mean (trueness) and standard deviation (precision) with the statistical results are reported in Table 1.

**Figure 5:**
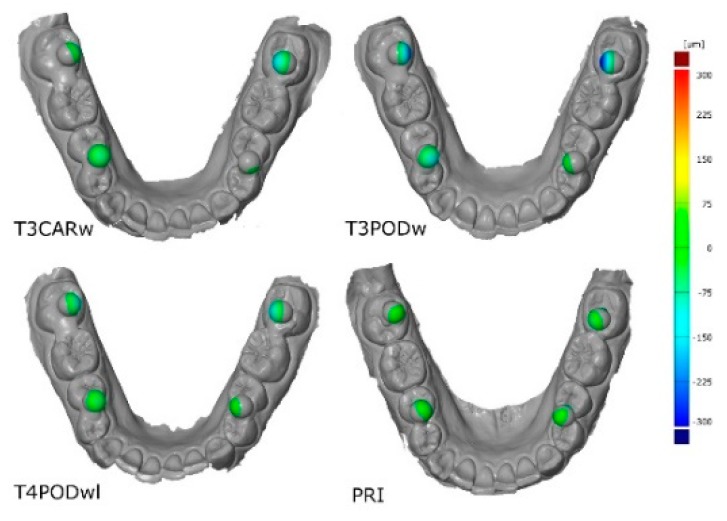
Graphical illustration for the typical three-dimensional arch distortion for the investigated intraoral scanners (IOS).

For a better overview, typical three-dimensional deviations for the different IOS are presented in Figure 5. Furthermore, the deviations in x-, y-, and z-direction are presented in Table 2.

The null hypothesis that the investigated IOS do not differ with regard to their transfer accuracy (trueness/precision) on full arch scans even in comparison to a conventional impression has to be partially rejected. 

## 4. Discussion

All investigated IOS hardware and software components used in this clinical study are currently available on the market. Before application, all IOS systems were updated with the latest software version and calibrated according to manufacturer’s specifications. To prevent any user influence, impression taking was performed by one experienced operator (L.K.) [30]. For better comparison of our results with those in the current literature, an established methodology was used [10]. Furthermore, results were reported for trueness and precision in accordance to ISO 5725 and as already described in other studies [10,17,28]. 

Previous studies have described the influence of scan path on the accuracy of full-arch scans [23,24,25]. Therefore, a predetermined scanning protocol was used, as recommended by Müller et al., who investigated different scan paths for the IOS Trios3 Pod [23]. In contrast, Passos et al. recently reported higher accuracy if using a more complex scan strategy for the two IOS devices (Primescan and Omnicam; Dentsply Sirona, Bensheim, Germany) [25]. However, it remains to be determined to what extent the practitioner has to learn different scanning paths or use an optimal scanning path for the respective scanner in dental practice. For better comparability of the IOS systems, a consistent scan path was maintained.

It is difficult to compare the results of this study with those in the literature because, to our knowledge, only two other studies have investigated full-arch impressions in patients using a reference [10,17]. Most studies superimposed datasets of digital scans and scanned models resulting from a conventional impression using a best-fit algorithm [9,23]. However, this setting only allows for a comparison of the two sources of digital data. They do not answer the question if the digital dataset matches the real patient situation. Furthermore, it remains uncertain whether any differences between two datasets are eliminated by using a compensation calculation such as the best-fit method [17]. O’Toole et al. investigated different alignment procedures and clearly recommended reference alignment to reduce measurement errors [20].

For the two short distances in the posterior segments (i.e., D1_2 and D3_4), more precise results were found using digital compared with conventional impressions. These findings are similar to those reported by Keul et al. [17]. In contrast, Ender et al. described the highest accuracy for the CVI technique, even for short distances [9]. However, more precise results for short-term spans are comparable with those of other studies because more accurate results for transfer accuracy were found within shorter distances. This could be explained by the matching or stitching error that increases with lengthening of the scan [31,32].

For longer distances, and also for those that completely cross the quadrant (D1_4), the CVI demonstrated more accurate results for trueness and precision. These findings are comparable with previous investigations [3,10]. However, the total deviations for the Trios 3 Pod and the Primescan in this clinical study were higher compared with the laboratory data reported by Ender et al. [9] and Iturrate et al. [33]. This could be due to different evaluation methods (percentiles) and furthermore explained by the in vivo conditions and the presence of saliva, oral structures, and patient movement(s) that may influence accuracy [34,35,36]. 

Overall, the latest scanners, Trios 4 and Primescan, delivered the most precise data for full-arch digital impressions. This, on the one hand, may be due to ongoing advances in hardware development, and/or updated software on the other. Especially for the Cerec systems, two studies demonstrated that the software version had significant impact on the accuracy of the IOS [9,19]. Other authors have indicated that current scanners would produce less error in relation to matching or stitching [20]. Findings from this study indicate that, at least for the Trios scanner, hardware also has a significant influence on the transfer accuracy of full-arch scans. From our findings, it may be hypothesized that the software of the scanning systems (Trios) probably uses a different algorithm(s). This may explain the high deviation in the data from the Trios 3 Pod. Whether this assumption is true is important information that manufacturers should provide. Alternatively, an invisible hardware problem of the handpiece may be the cause of the difference [26]. 

In summary, the results of the present study demonstrated that, for short distances up to a quadrant, current IOS systems yield less deviation compared with conventional impressions. With regard to full-arch impressions, it depends on the definition or indication whether an intraoral scanner can be used for full-arch impressions. Further studies with larger numbers of subjects are needed. Nevertheless, the data presented showed that the most recent scanners have improved with regard to accuracy of full arch scans.

## 5. Conclusions

Within the limitations of this clinical study, current IOS scanners equipped the with latest software versions demonstrated less deviation for short-span distances (D1_2 and D3_4) compared with CVI techniques. However, for long-span distances, the CVI technique provided the lowest deviation, although no significant difference was demonstrated for PRI and T4PODwl. Hardware components of the Trios scanner exhibited an influence on transfer accuracy.

## Figures and Tables

**Table 1:** Deviations (Mean ± standard deviation (SD) (µm)) of the linear distances (D1_2, D1_3, D1_4, D2_3, D2_4, D3_4) and statistical analysis for trueness (upper right part) and precision (presented in bold type) according to International Organization for Standardization (ISO) 5725.

Linear Distances	Impression Technique		*p* Value				
		Mean (Trueness) ±SD (Precision) (µm)	T3CARw	T3PODw	T4PODwl	PRI	CVI
D1_2	T3CARw	21.6 ± 12.6	-	0.999	0.997	0.776	0.414
	T3PODw	19.4 ± 13.4	**0.664**	-	>0.999	0.924	0.322
	T4PODwl	18.0 ± 21.7	**0.363**	**0.500**	-	0.993	0.558
	PRI	13.8 ± 8.4	**0.751**	**0.308**	**0.221**	-	0.021 *
	CVI	33.5 ± 6.3	**0.362**	**0.108**	**0.120**	**0.268**	-
D1_3	T3CARw	112.6 ± 83.0	-	0.026 *	0.948	0.348	0.229
	T3PODw	294.4 ± 49.6	**0.351**	-	0.001 *	0.001 *	0.001 *
	T4PODwl	84.8 ± 32.0	**0.131**	**0.333**	-	0.084	0.029 *
	PRI	32.1 ± 13.6	0.041 *	0.042 *	**0.093**	-	0.379
	CVI	15.5 ± 13.8	0.039 *	0.040 *	**0.090**	**0.857**	-
D1_4	T3CARw	247.4 ± 203.0	-	0.180	0.694	0.403	0.300
	T3PODw	515.0 ± 100.3	**0.163**	-	0.001 *	0.001 *	0.001 *
	T4PODwl	120.6 ± 70.6	**0.070**	**0.383**	-	0.600	0.234
	PRI	64.2 ± 47.1	0.032 *	**0.089**	**0.206**	-	0.808
	CVI	36.3 ± 32.6	0.021 *	0.034 *	**0.051**	**0.525**	-
D2_3	T3CARw	20.6 ± 22.0	-	<.001 *	0.298	0.998	0.907
	T3PODw#	152.6 ± 23.6	**0.722**	-	0.002 *	<0.001 *	0.001 *
	T4PODwl#	54.2 ± 27.1	**0.977**	**0.822**	-	0.393	0.107
	PRI	24.8 ± 21.8	**0.887**	**0.788**	**0.969**	-	0.724
	CVI	12.3 ± 3.8	<0.001 *	**0.067**	**0.066**	0.001 *	-
D2_4	T3CARw	87.2 ± 79.2	-	0.062	>0.999	0.854	0.514
	T3PODw	233.4 ± 55.5	**0.292**	-	0.016 *	0.003 *	0.004 *
	T4PODwl	94.0 ± 44.1	**0.107**	**0.356**	-	0.484	0.105
	PRI	47.7 ± 42.0	**0.090**	**0.325**	**0.914**	-	0.802
	CVI	26.0 ± 13.9	0.005 *	0.005 *	**0.084**	0.022 *	-
D3_4	T3CARw	18.8 ± 14.4	-	0.898	>0.999	>0.999	0.977
	T3PODw	12.4 ± 8.0	**0.** **194**	-	0.863	0.831	0.562
	T4PODwl	19.4 ± 14.2	**0.832**	**0.394**	-	>0.999	0.985
	PRI	20.5 ± 15.5	**0.836**	**0.146**	**0.696**	-	0.996
	CVI	23.9 ± 14.4	**0.986**	**0.210**	**0.846**	0.825	-

Significant differences (*p* value <0.05) are highlighted with an asterisk (*). Not normally distributed (#).

**Table 2:** Results for deviations in x-, y-, and z-direction for the different spheres and the investigated IOS.

Impression Technique	Patient	*Sphere 1*			*Sphere 2*			*Sphere 3*			*Sphere 4*		
		x	y	z	x	y	z	x	y	z	x	y	z
**T3CARw**	1	0.127	0.094	0.024	0.028	0.067	0.051	−0.075	−0.016	0.005	−0.115	−0.175	0.056
	2	0.086	0.034	0.008	0.003	0.025	0.074	−0.011	0.03	−0.025	−0.083	−0.086	0.059
	3	0.016	0.03	0.005	−0.001	−0.006	0.068	−0.004	−0.005	−0.019	−0.015	−0.021	0.056
	4	−0.195	−0.19	0.001	0.062	−0.067	0.046	0.058	−0.012	0.02	0.182	0.204	0.054
	5	0.02	−0.005	−0.005	0.026	0.004	0.073	−0.019	0.037	−0.007	−0.026	−0.046	0.047
T3PODw	1	0.24	0.215	0.028	0.023	0.089	0.051	−0.097	−0.041	−0.015	−0.219	−0.256	0.069
	2	0.201	0.186	0.023	0.03	0.087	0.088	−0.075	−.042	−0.014	−0.213	−0.223	0.083
	3	0.149	0.179	0.042	0.005	0.072	0.055	−0.071	−0.054	0.019	−0.12	−0.156	0.067
	4	0.207	0.214	0.093	0.006	0.061	0.053	−0.088	−0.052	0.017	−0.128	−0.195	0.053
	5	0.219	0.195	0.063	0.026	0.083	0.044	−0.085	−0.02	0.025	−0.155	−0.233	0.074
T4PODwl	1	0.076	0.054	0.048	0.016	0.032	−0.003	−0.043	−0.013	0.056	−0.044	−0.077	0.004
	2	0.09	0.074	0.033	0.011	0.039	0.053	−0.028	−0.014	0.009	−0.101	−0.083	0.046
	3	0.024	0.024	0.039	0.016	−0.004	0.003	−0.012	0.016	0.05	−0.042	−0.042	0.017
	4	0.025	0.026	0.052	0.003	0.001	0.001	-0.007	−0.006	0.046	−0.018	−0.028	0.017
	5	0.061	0.04	0.028	0.025	0.032	0.037	−0.02	0.001	0.01	−0.079	−0.052	0.028
PRI	1	−0.005	−0.015	0.006	−0.002	0.01	0.036	−0.001	0.003	−0.001	0.005	0.008	0.023
	2	0.006	0.023	0.019	0.037	0.018	0.022	−0.018	−0.006	0.019	−0.052	−0.01	0.026
	3	−0.033	−0.021	0.014	0.01	0.003	0.017	−0.001	−0.011	0.02	0.014	0.04	0.032
	4	0.046	0.046	0.044	0.024	0.017	0.001	−0.047	0.018	0.04	−0.037	−0.058	0.02
	5	−0.018	−0.025	−0.019	0.027	0.008	0.088	-0.001	0.015	−0.033	−0.015	0.008	0.079
